# Lysosomal free sialic acid storage disorder iPSC-derived neural cells display altered glycosphingolipid metabolism

**DOI:** 10.1038/s41598-025-12682-4

**Published:** 2025-08-13

**Authors:** Marya S. Sabir, Vukasin M. Jovanovic, Seungmi Ryu, Chaitali Sen, Pinar Ormanoglu, Laura Pollard, Richard Steet, William A. Gahl, Marjan Huizing, Carlos A. Tristan, Frances M. Platt, May Christine V. Malicdan

**Affiliations:** 1 UDP Translational Laboratory, NIH Undiagnosed Diseases Program, National Human Genome Research Institute, National Institutes of Health, Bethesda, MD USA; 2 NIH Oxford-Cambridge Scholars Program, University of Oxford, Oxford, UK; 3 Stem Cell Translation Laboratory, National Center for Advancing Translational Sciences, National Institutes of Health, Rockville, MD USA; 4 Greenwood Genetic Center, Greenwood, SC USA; 5 Human Biochemical Genetics Section, Medical Genetics Branch, National Human Genome Research Institute, National Institutes of Health, Bethesda, MD USA; 6 Department of Pharmacology, University of Oxford, Oxford, UK

**Keywords:** Salla disease, Sialin, Glycosphingolipids, Gangliosides, Sialidase, Astrocytes, Molecular neuroscience, Neural stem cells, Mechanisms of disease

## Abstract

Lysosomal free sialic acid storage disorder (FSASD) is a rare neurodegenerative disease caused by biallelic mutations in *SLC17A5*, encoding the lysosomal sialic acid exporter, SLC17A5 (sialin). While the involvement of oligodendroglia in FSASD pathogenesis is established, the roles of other neural cell types remain elusive. In this study, we utilized radial glial cells (iRGCs), immature and mature astrocytes (iIAs and iMAs, respectively), and cortical neurons (iCNs) differentiated from induced pluripotent stem cells (iPSCs) derived from two individuals with FSASD, alongside two independent healthy donors for comparison. We employed a multifaceted profiling approach, including the assessment of cellular glycosphingolipids (GSLs), transcriptomics focused on GSL metabolism genes, and 4-methylumbelliferone-based lysosomal enzyme activity measurements. Our findings revealed significant elevations in free sialic acid levels across all FSASD cell types, indicating that iPSCs and derived iRGCs, iIAs, iMAs and iCNs may be used to model FSASD in vitro. We observed significant alterations in the abundance of specific GSL species, predominantly in mature astrocytes, with fewer changes in other cell types. Transcriptomic analyses uncovered differential expression of genes involved in GSL catabolism, including those encoding glycohydrolases. Enzyme assays corroborated the transcriptomic findings, showing heightened glycohydrolase activities, particularly in mature astrocytes. Collectively, these data may help refine our understanding of neural cell phenotypes and potential contributors to selective vulnerability in FSASD.

## Introduction

Lysosomal free sialic acid storage disorder (FSASD) is a rare autosomal recessive, neurodegenerative disease caused by biallelic variants in *SLC17A5*^1–3^, which encodes sialin, a lysosomal membrane transporter responsible for the efflux of sialic acid and other acidic hexoses from the lysosome to the cytosol^4–8^. Mutations in *SLC17A5* result in the accumulation of excessive unconjugated “free” sialic acid in lysosomes within cells of multiple tissues, including the central nervous system^7,9,10^. Clinically, FSASD encompasses a spectrum ranging from a severe infantile-onset form (infantile sialic acid storage disease or ISSD; MIM#269,920) to a milder, slowly progressive neurodegenerative form (Salla disease, MIM#604,369)^1–3,11,12^.

The neurological manifestations of FSASD— including developmental delay, spasticity, athetosis, seizures, and cognitive impairment^11,12^—indicate a widespread disruption of homeostasis in the brain. On brain MRI, affected individuals present predominantly with hypomyelination, corpus callosum hypoplasia, and progressive cerebellar atrophy^11,13–17^. It is well established that neurons and glial cells rely on precise metabolic regulation and lipid homeostasis to maintain their function and survival^18^. While prior studies have highlighted the role of oligodendroglia, particularly in relation to the hypomyelination observed in FSASD^16,19^, the contributions and potential vulnerabilities of other neural cell types remain poorly understood.

FSASD is part of a broader group of lysosomal storage disorders (LSDs), including GM1 and GM2 gangliosidoses, Gaucher disease, and Niemann-Pick disease, all of which are characterized by lysosomal dysfunction and the accumulation of undegraded substrates^20^. Many of these disorders exhibit disruptions in glycosphingolipid (GSL) metabolism, a pathway essential for neuronal differentiation^21^ and synaptic function^22^. Gangliosides, i.e., sialylated glycosphingolipids (GSLs), are the predominant glycoconjugates on the surfaces of vertebrate nerve cells and play a critical role in maintaining myelin stability and axon structure^23,24^. Previous studies have shown reduced turnover of sialoglycoconjugates in FSASD fibroblasts^25–31^, however, the full extent of GSL dysregulation in neural cells and its potential role in FSASD remains unclear.

In this study, we sought to profile the GSL levels across various neural cell types derived from FSASD iPSCs, with the goal of identifying cell-type-specific vulnerabilities. We utilized radial glial cells (iRGCs), immature and mature astrocytes (iIAs and iMAs), and cortical neurons (iCNs) derived from iPSCs of individuals with FSASD and compared them to neural cells from healthy donors. Using a comprehensive profiling approach—including GSL quantification, transcriptomic analysis of GSL metabolism genes, and lysosomal enzyme activity assays—we sought to elucidate the potential impact of sialin deficiency on GSL metabolism across distinct neural lineages. Our findings reveal significant metabolic alterations in astrocytes, particularly in iMAs, emphasizing their potential role in FSASD pathogenesis and identifying putative therapeutic targets.

## Results

**Fig. 1:**
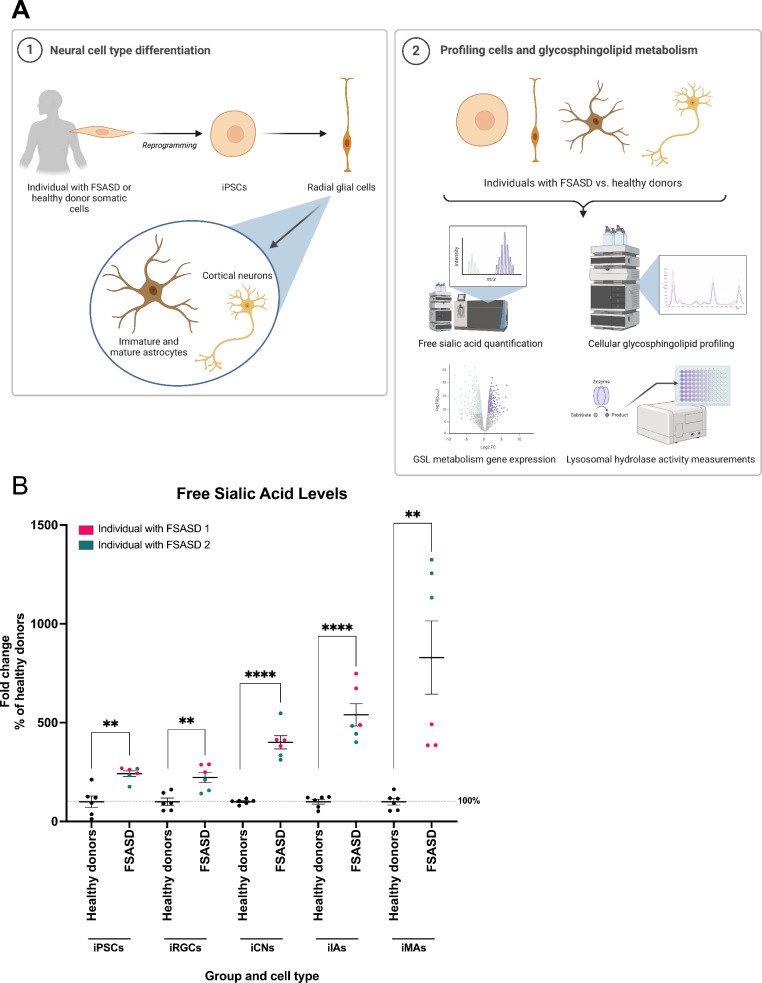
Schematic overview of study and free sialic acid levels across cell types using whole-cell lysates. (**A**) Two individuals with FSASD and two healthy donor somatic cell lines were reprogrammed to iPSCs and differentiated to radial glial cells (iRGCs). iRGCs were further differentiated to immature and mature astrocytes (iIAs and iMAs, respectively) as well as cortical neurons (iCNs). All cell types were then profiled using several assays including free sialic acid quantification, glycosphingolipid (GSL) quantification, examination of genes involved in GSL metabolism, and measurement of lysosomal GSL hydrolase activities. Figure created using BioRender.com. (**B**) Free sialic acid levels quantified via UPLC-MS/MS were normalized to total protein content in iPSCs, iRGCs, iCNs, iIAs, and iMAs and depicted as percent of healthy donors. Mean ± SEM; unpaired t-test per cell type with *p*-value < 0.005 (**) and < 0.0005 (****).

We utilized iPSC-derived neural cell types from two individuals with FSASD, each harboring distinct *SLC17A5* variants, and compared them to cells from two healthy donors (Supplementary Table 1). A multifaceted approach was employed to comprehensively profile these cells (Fig. 1A).

### Free sialic acid is elevated and GSL profiles are altered in FSASD neural cell types

We utilized UPLC-MS/MS to quantify free sialic acid levels across all cell types included in our study. Compared to the healthy donor group, we observed elevated free sialic levels in FSASD across all cell types, with iPSCs (242%), iRGCs (223%), iCNs (400%), iIAs (540%), and iMAs (830%) showing progressively higher levels (Fig. 1B). Collectively, these results demonstrate the presence of the primary biochemical defect in FSASD across all FSASD cell types, signifying their potential as in vitro models for studying this disorder.

**Fig. 2:**
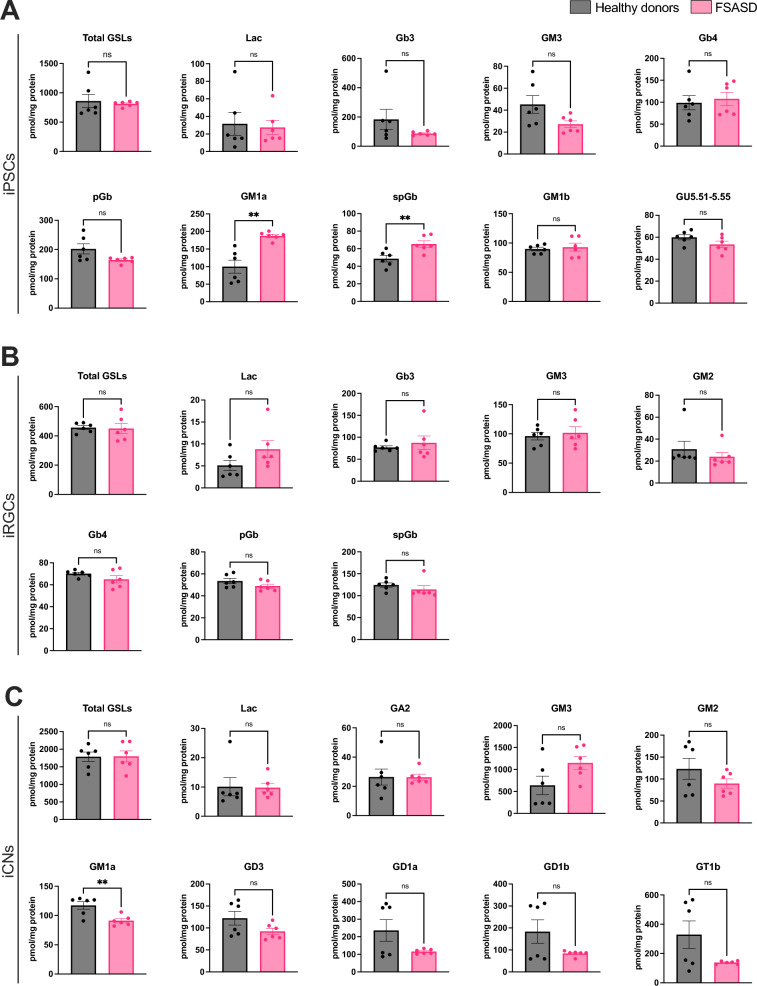
Levels of total GSLs and individual GSL species in FSASD and healthy donor iPSCs, iRGCs, and iCNs. (**A**) iPSCs, (**B**) iRGCs, and (**C**) iCNs. GSL levels quantified via HPLC were normalized to total protein content of the whole cell lysates. Each point corresponds to one of the three replicates for each cell line. Mean ± SEM; unpaired t-test *p*-value < 0.005 (**), ns = not significant. GU = glucose unit.

Next, we used sensitive and quantitative normal-phase high-performance liquid chromatography (HPLC) assays, to examine GSL levels in each cell type. Compared to healthy donors, FSASD iPSCs exhibited elevated GM1a and spGb levels, with no changes in total GSLs or other species, although GM3 and pGb showed a trending decrease (Fig. 2A). In FSASD iRGCs, total GSLs and all detected species were similar between healthy donors and FSASD (Fig. 2B). In contrast, iCNs displayed a reduction in GM1a levels, along with borderline decreases in GM2, GD3, GD1a, GD1b, and GT1b (Fig. 2C). Three of the four major brain gangliosides^32^, i.e., GD1a, GD1b and GT1b, displayed considerable variation in healthy donor cortical neurons while FSASD cells showed significantly less variation (Fig. 2C).

**Fig. 3:**
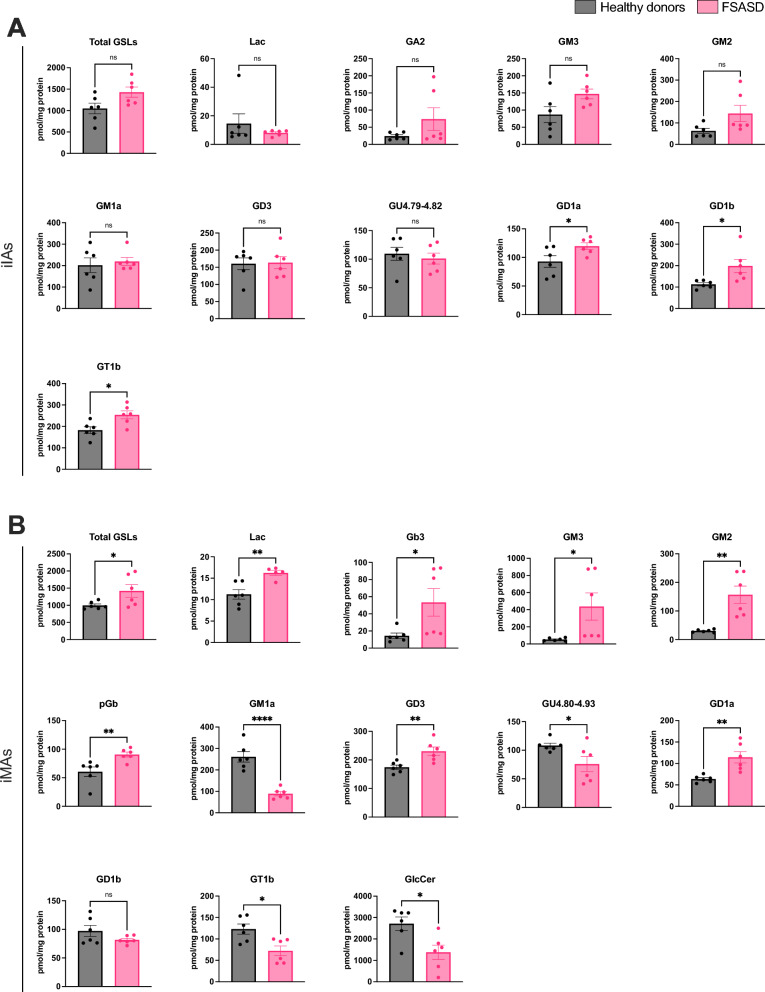
Levels of total GSLs and individual GSL species in FSASD and healthy donor iIAs and iMAs. (**A**) iIAs and (**B**) iMAs. GSL levels quantified via HPLC were normalized to total protein content of the whole cell lysates. Each point corresponds to one of the three replicates for each cell line. Mean ± SEM; unpaired t-test *p*-value < 0.05 (*), < 0.005 (**), < 0.00005 (****), ns = not significant. GU = glucose unit.

Among the examined cells, FSASD iIAs and iMAs showed the greatest levels of GSL aberrations compared to healthy donors. FSASD iIAs displayed elevated levels of GD1a, GD1b, and GT1b (Fig. 3A). Total GSLs, GM3, and GM2 showed a borderline significant upward trend (Fig. 3A), while other detected species in iIAs, including Lac, GM1a, GD3, and GU4.79–4.82 did not exhibit changes (Fig. 3A). In contrast, FSASD iMAs displayed elevated total GSLs, Gb3, GM3, GM2, pGb, GD3, GD1a, and Lac, while GM1a, GU4.80–4.93, GT1b, and glucosylceramide (GlcCer) were decreased (Figs. 3B). GD1b was unchanged in FSASD iMAs (Figs. 3B). Supplementary Figs. 1 and 2 present individual-level GSL profiles, while Supplementary Fig. 3A and B display representative HPLC traces for GSLs and GlcCer in iMAs, respectively. Raw peak area values for all samples are also provided in Supplementary Table 3.

Interestingly, only FSASD iMAs exhibited elevated levels of total GSLs, GM3, and GM2 (Figs. 3B and 7). GM1a levels were elevated in FSASD iPSCs, but decreased in iCNs and iMAs (Figs. 2, 3, 7). Free sialic acid levels correlated with elevated levels of gangliosides only in FSASD iPSCs and derived iIAs; no significant correlations were observed in the other cell types (Supplementary Fig. 4).

### GSL catabolism genes are altered primarily in FSASD mature astrocytes

GSL catabolism occurs in the lysosome and is dependent on the action of several hydrolases that retain specificity for certain substrates and glycan linkages^33^. Due to the changes observed in GSL abundance in the FSASD iPSC-derived neural cell types, we performed bulk transcriptomic analyses to examine GSL metabolism genes, including those involved in catabolism.

**Fig. 4:**
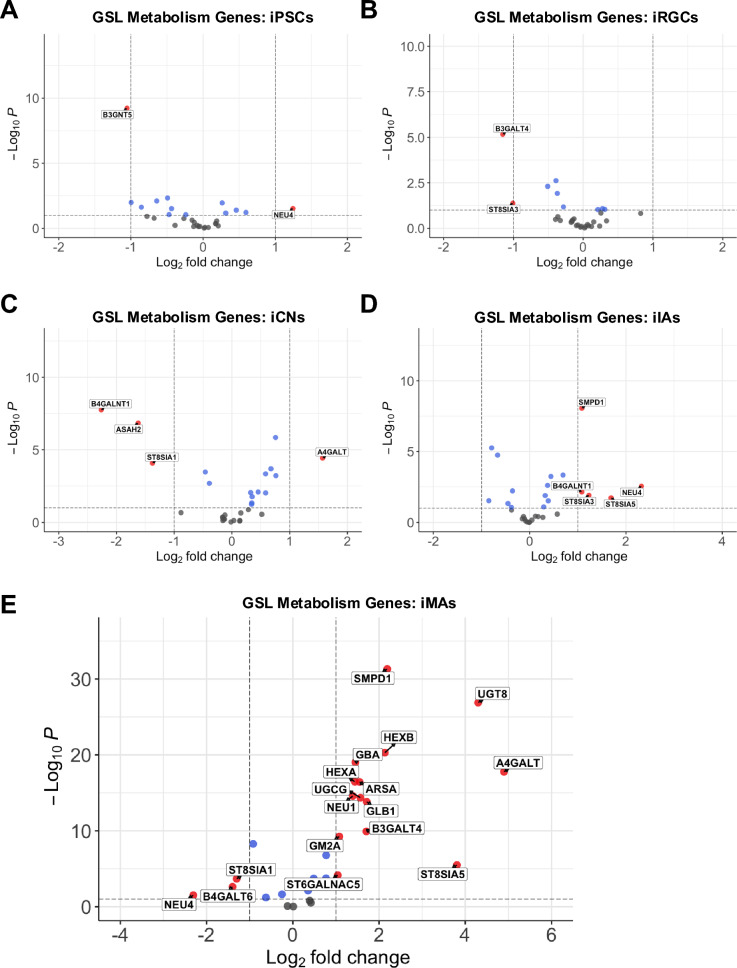
Expression of GSL metabolism genes across cell types. GSL biosynthesis and catabolism gene expression in (**A**) iPSCs, (**B**) iRGCs, (**C**) iCNs, (**D**) iIAs, and (**E**) iMAs. Log_2_ fold change cut-off set at 1 and adjusted -log_10_ *p*-value cut-off set at 10^–2^. Red circles (with gene names annotated) indicate genes up or down-regulated meeting the indicated *p*-value and log_2_ fold-change cut-offs. Blue circles indicate genes meeting the *p*-value cut-off only. Gray circles indicate genes not significantly altered. Refer to Supplementary Table 2 for GSL metabolism gene expression across each cell type. The gene list was derived from Platt^42^ where the associated GSL metabolic pathways are depicted.

Sialidases (i.e., neuraminidases) mediate the removal of sialic acids from glycoconjugates^34^. One of the mammalian neuraminidases, *NEU4* (encoding NEU4 sialidase)^34–37^, localizes to lysosomes and other organelles, exhibited elevated transcriptional expression in FSASD iPSCs and iIAs, but decreased expression in iMAs (Fig. 4A, D, E). Conversely, *NEU1* (encoding for lysosomal NEU1 sialidase)^38,39^, had elevated expression only in iMAs (Fig. 4E). *NEU3* encoding NEU3, predominantly localizes to the plasma membrane^38^, was unchanged in FSASD cells (Fig. 4); however, in vivo studies have demonstrated its involvement in lysosomal ganglioside catabolism^40,41^, indicating functional relevance within the lysosomal compartment. *NEU2* encoding NEU2, a cytosolic sialidase^38^, similarly exhibited no changes in expression in FSASD cells (Fig. 4).

Expression of genes encoding lysosomal glycohydrolases (β-hexosaminidase, β-glucocerebrosidase, β- and α-galactosidases) involved in GSL catabolism^33^ were mostly upregulated in FSASD. β-hexosaminidase forms three isoenzymes (HexA, HexB, HexS) from α (*HEXA*) and β (*HEXB*) subunits. In FSASD iMAs, expression of both *HEXB* and *GM2A*, which encodes the GM2 activator protein that assists HexA in GM2 degradation, was elevated (Fig. 4E). Expression of *GBA1* (encodes β-glucocerebrosidase), which hydrolyzes GlcCer and is associated with Gaucher disease^43^ was elevated (Fig. 4E). *GLB1* (encodes β-galactosidase, the enzyme deficient in GM1 gangliosidosis^44^) expression was also elevated (Fig. 4E). Lastly transcript levels of *GLA*, a gene that encodes α-galactosidase and is responsible for Fabry disease^45^, were not altered in FSASD neural cells (Fig. 4). The full dataset of GSL metabolism gene expression results is available in Supplementary Table 2.

### Lysosomal enzyme activity measurements validate transcriptomic dysregulation of several hydrolases

**Fig. 5:**
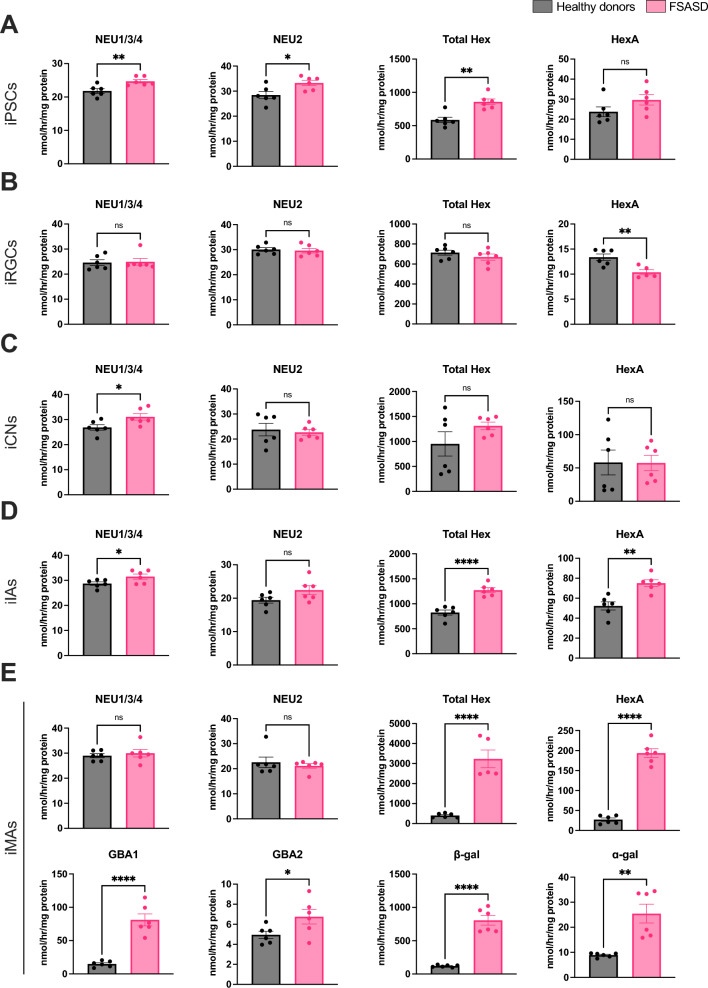
Activity levels of GSL hydrolases in the neural cell types. (**A**) iPSCs, (**B**) iRGCs, (**C**) iCNs, (**D**) iIAs, and (**E**) iMAs. The following enzyme activities were measured via 4-MU-based assays in all cell types (using whole cell lysates): neuraminidase (NEU1/3/4 and NEU2) and β-hexosaminidase (total Hex and HexA). Additional enzymes were examined in iMAs including glucocerebrosidase (GBA1 and GBA2), β-galactosidase (β-gal), and ɑ-galactosidase (ɑ-gal). Activity represented as nmol per hour per mg total protein content. Each point corresponds to one of the three replicates for each cell line. Mean ± SEM; unpaired t-test* p*-value < 0.05 (*), < 0.005 (**), < 0.00005 (****), ns = not significant.

To validate the transcriptomics findings, we employed fluorescent 4-methylumbelliferone (4-MU) assays to quantify neuraminidase activities, including NEU1/3/4 and cytosolic NEU2. In FSASD iPSCs, iCNs, and iIAs, NEU1/3/4 activity was elevated (Fig. 5A, C, D). NEU2 activity was only slightly elevated (117% of healthy donors) in FSASD iPSCs (Fig. 5A).

We conducted two 4-MU assays to quantify β-hexosaminidase activities, including total and HexA. In FSASD iPSCs, and particularly in iIAs and iMAs, total Hex was elevated when compared to healthy donors (Fig. 5A, D, E). HexA was elevated only in FSASD iIAs and iMAs (Fig. 5D, E). Conversely, HexA activity levels were decreased in FSASD iRGCs (Fig. 5B). In iCNs, total Hex and HexA activities displayed considerable variation in healthy donors while FSASD cells showed some but less variation (Fig. 5C).

**Fig. 6:**
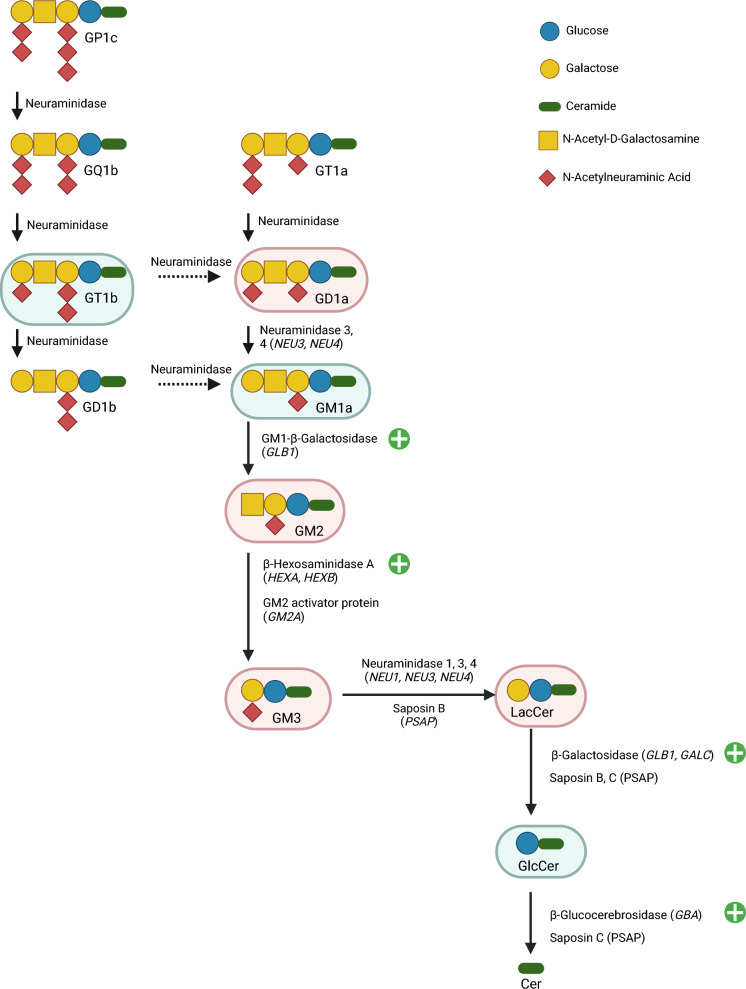
Simplified representation of GSL catabolism and levels in FSASD iMAs. The enzymes responsible for catabolism and co-factors are listed next to each arrow. Sialic acid moieties are denoted in red diamond shapes. The species that are elevated (red oval shading) or decreased (green oval shading) in individuals with FSASD compared to healthy donors are indicated; not all species depicted are detected in iMAs and total GSLs, Gb3, pGb, and GD3 are also elevated in FSASD iMAs. GD1b is not altered in FSASD iMAs. Green circles containing crosses indicate elevated hydrolase activity levels. Figure adapted from^42^ and created using BioRender.com.

**Fig. 7:**
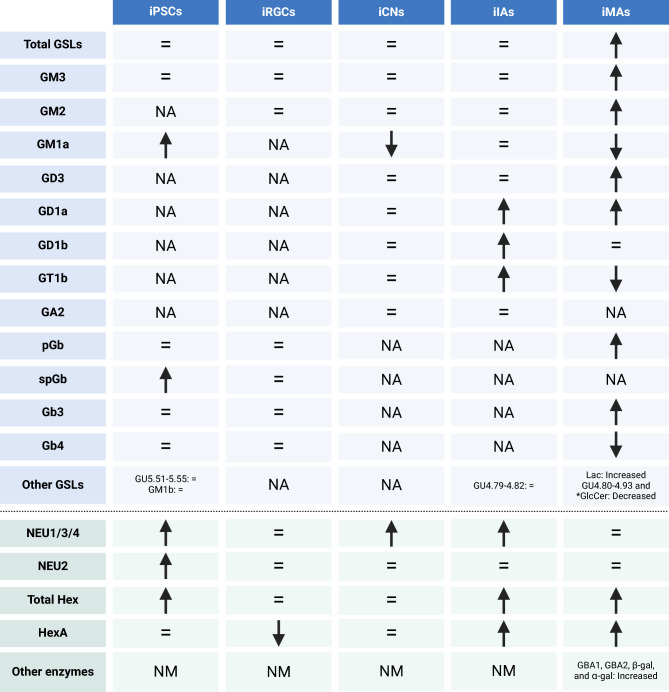
Summary of GSL and enzyme activity levels across each cell type. Comparison of individuals with FSASD versus healthy donors. Up arrows denote significantly elevated GSL species or enzyme activities, while down arrows indicate significantly decreased levels or activities. An equal sign signifies no significant difference between FSASD and healthy donor groups. *GlcCer was assessed only in iMAs. NA (not applicable) indicates that the GSL was not detected in that cell type. NM (not measured) means that no additional enzyme activity assays were conducted for that cell type.

Due to the increased transcriptional expression of *GBA1* and *GLB1* observed in FSASD iMAs, we measured the activities of these hydrolases, distinguishing the lysosomal GCase (GBA1) and non-lysosomal GBA2 activities using the inhibitor *N*-butyldeoxygalactonojirimycin^46,47^. GBA1, GBA2, and GLB1 activities were increased in FSASD cells compared to healthy donors (Fig. 5E). Interestingly, α-galactosidase activity was elevated in FSASD as a group, but the two individuals with FSASD showed varying activity levels (Fig. 5E), which correlated with their Gb3 levels. In other words, FSASD individual 1 retained a higher level of Gb3 compared to FSASD individual 2 and also showed a greater increase in α-galactosidase activity (Figs. 3B and 5E). Figure 6 provides a schematic of GSL catabolism, highlighting adaptations based on FSASD iMA findings. Figure 7 summarizes the enzyme activity results, while Supplementary figure 5 presents individual-level enzyme activities.

## Discussion

This study offers preliminary insights into neural cell phenotypes and potential mechanisms that may underlie selective vulnerability in FSASD. Our findings reveal that free sialic acid accumulation occurs in all FSASD-derived cells, with astrocytes exhibiting selective vulnerability—followed by cortical neurons—to disrupted GSL homeostasis, a key feature of several lysosomal storage disorders^48^.

### Astrocyte-specific vulnerability in FSASD

Among all cell types examined, iMAs displayed the highest levels of free sialic acid accumulation. As metabolically active cells, astrocytes play a vital role in supporting neuronal energy requirements by shuttling glycolytic derived metabolites^49^. Impaired sialic acid metabolism within astrocytes could not only impair their intrinsic function but also alter the interaction of astrocytes with neurons^50^ and other glial cells^51^.

The brain contains 10-30-fold more gangliosides than any human tissue, with gangliosides representing 80% of all glycans and over 75% of the brain’s total sialic acid content^22,52^. Previously, we reported a significant increase in gangliosides in the CSF of individuals with FSASD^51^. Our current study extends these findings by revealing cell-type-specific alterations in GSL abundance, with astrocytes demonstrating the most significant dysregulation. Interestingly, iRGCs exhibited no remarkable alterations, suggesting that they either possess a unique metabolic phenotype or compensate for sialic acid accumulation differently than other neural cell types.

Astrocytes, the most abundant cell type in the CNS^53^, play a crucial role in neuroinflammation by regulating CNS infiltration by pro-inflammatory leukocytes^54–57^. In FSASD iMAs, we identified an elevation in LacCer levels, a GSL produced by astrocytes during chronic CNS inflammation that amplifies inflammation and promotes neurodegeneration^57^. The increase in LacCer may suggest a mechanistic link between astrocyte dysfunction and inflammatory signaling, positioning LacCer metabolism as a potential therapeutic target for FSASD and related neurodegenerative diseases.

FSASD-derived iIAs showed significant accumulation of several GSL species, including three of the four major brain gangliosides—GD1a, GD1b, and GT1b—that together constitute over 90% of the brain ganglioside mass^32^. When iIAs are compared to iMAs, there was a notable shift in ganglioside composition: the levels of GM1a and GT1b decreased, while GD1a levels increased, and GD1b remained unchanged. The alteration in GSL profiles during terminal astrocyte differentiation suggests that dysregulated GSL levels in FSASD may impair astrocyte maturation.

More broadly, although the extent of GSL accumulation observed in FSASD-derived cells is modest compared to the pronounced elevations of GM1/GA1 and GM2 observed in classic GM1^58^ and GM2^59^ gangliosidoses, respectively, subtle shifts in GSL homeostasis in the CNS may carry functional consequences. In the CNS, disruptions in sphingolipid (including GSL) composition can influence membrane microdomain organization^60^, protein trafficking^61^, cell–cell recognition^62^ as well as modulate astrocyte-mediated inflammatory signaling^63^, These observations raise the possibility that modest GSL alterations in FSASD could contribute to disease pathogenesis and merits further mechanistic investigation.

### Implications of GSL catabolism in FSASD

Pathogenic variants in specific ganglioside glycosidases are implicated in several lysosomal storage diseases with neurological manifestations, such as the GM2 and GM1 gangliosidoses, caused by mutations in *HEXA/HEXB* and *GLB1* genes, respectively^64^. In FSASD, despite increased *HEXB* expression and elevated total Hex and HexA activity, GM2 accumulated in iMAs, suggesting an insufficient compensatory response. In contrast, elevated *GLB1* expression and β-galactosidase activity correlated with decreased GM1a levels. Furthermore, upregulated *GBA1* expression and enzyme activity in FSASD iMAs led to reduced GlcCer levels despite the accumulation of upstream GSL species. However, it remains unclear whether this glucosylceramide pool was depleted through conversion to glucosylsphingosine in these models. Collectively, these findings suggest that iMAs may serve as a model for investigating GSL catabolism and its potential therapeutic modulation.

### Linking altered sialic acid and ganglioside levels

The correlation of elevated free sialic levels with elevated ganglioside levels in FSASD iPSCs and iIAs may appear paradoxical, given that lysosomal sialic acid recycling is impaired in FSASD. However, excessive free sialic acid in lysosomes could lead to secondary ganglioside storage, as seen in other lysosomal diseases, since free sialic acid acts as a competitive inhibitor of lysosomal sialidases^38,65^. More broadly, changes in the intra-lysosomal environment, particularly pH, resulting from sialin deficiency and the accumulation of unconjugated free sialic acid, may reduce proton and acidic sugar trafficking, impairing digestion of macromolecules by other lysosomal enzymes, which require an optimal acidic pH to function effectively^66^. While sialin functions as a proton-driven transporter with pH-dependent activity^7,10,67^, further investigation into potential pH alterations in FSASD cells is required.

### GM1a as a potential therapeutic target

The elevated level of GM1a in iPSCs, followed by its subsequent decline in iCNs and iMAs, is particularly noteworthy as GM1a is purported to be neuroprotective^52^. GM1a levels increase during neuronal development, regulating calcium flux across the nuclear membrane and interacting with proteins including nerve growth factor, brain-derived neurotrophic factor, and glial-derived neurotrophic factor via Trk tyrosine kinases to promote signaling^33,68–70^. Since GM1a predominates in myelin and adult oligodendrocytes^71,72^, and given their compromised state in FSASD, strategies to increase GM1a levels, such as exogenous administration^52^, may offer a potential therapeutic strategy. Recent studies have shown that the GM1 oligosaccharide head group, the bioactive component of GM1, enhances neuronal survival and protects against mitochondrial oxidative stress in dopaminergic and glutamatergic primary cultures, suggesting its potential as a neuroprotective agent in Parkinson’s disease models, a neurodegenerative disorder also linked to lysosomal dysfunction^73^. Terminally differentiated FSASD cell types, such as iCNs and iMAs, are likely the most suitable cell models for evaluating GM1a-based interventions.

### Limitations

While this study offers insights into FSASD neural cell pathology, we acknowledge several limitations. First, the two FSASD iPSC lines used in this study were derived from individuals with different genetic backgrounds and clinical severities, without isogenic controls, which limits our ability to distinguish disease-related changes from patient-specific variability. Future studies using isogenic models will be critical to disentangle genotype-specific effects. Second, while the study sample size is limited, each line was independently differentiated and underwent comprehensive biochemical and molecular profiling. Given the significant time and resources required for iPSC maintenance, neural differentiation, and downstream assays, this study was designed as a focused, in-depth investigation to establish a framework for studying FSASD. Additional patient-derived and isogenic lines will be essential to validate and expand upon these findings.

Third, developmental stage influences brain GSL composition, as shown in mice^74,75^, rats^76–78^, and human^79,80^, and differences in donor age (1.3 and 4 years for FSASD; 0 years for healthy donors) may contribute to baseline variation in GSL profiles. Furthermore, iPSC-derived models may not fully capture the complexity of primary neural tissue, limiting direct translation to in vivo pathology. Nevertheless, these cell models provide a valuable platform for exploring disease-relevant mechanisms and generating hypotheses for future validation in more physiologically complex systems, such as FSASD mouse models^19,81–83^. Future studies could incorporate oligodendroglial cells, co-culture systems, or organoid models to better capture GSL alterations.

Finally, our conclusions regarding disrupted GSL homeostasis, particularly in mature astrocytes, may remain speculative and warrant further mechanistic investigation. Detailed molecular analysis, including transcriptomic and proteomic profiling, will be essential to clarify if the downstream consequences of sialin deficiency align in vitro findings with the human disease.

Despite these limitations, this study is the first to comprehensively profile GSL metabolism in FSASD neural cell types, highlighting astrocytes as particularly susceptible and offering novel cellular models for high-throughput screening of disease-modifying pharmacologic agents. Altogether, developing treatments specifically targeting astrocytes, including gene therapy approaches to correct sialin function, presents a promising new avenue for therapeutic intervention to alleviate neurological symptoms in individuals with FSASD.

## Methods

### Study subjects and ethical approvals

FSASD individual 1 was enrolled in National Institutes of Health (NIH) protocol 14-HG-0071 (NCT02089789), which received approval from the NIH National Human Genome Research Institute Institutional Review Board. Fibroblasts from FSASD individual 2 were sourced from the Biobank Unit of the Finnish Institute for Health and Welfare (THL) in Helsinki, Finland, with approval from the THL Institutional Review Board. The fibroblasts were subsequently transferred to the NIH under a material transfer agreement.

iPSC lines from Healthy Donor 1 and 2, NCRM-5 and LiPSC-GR1.1 respectively, were generated by and obtained from the NIH Regenerative Medicine Program (https://commonfund.nih.gov/stemcells/stem-cell-lines-scl).

### iPSC reprogramming and characterization

Fibroblasts derived from individuals with FSASD were reprogrammed and clonally selected using episomal plasmids to induce the ectopic expression of OCT4, SOX2, KLF4, LMYC, and Lin28 genes (service provided by ALSTEM, Inc.)^84^. The resulting iPSCs were thoroughly characterized to confirm their utility and fidelity, as detailed in our previously published study of these cell lines^84^.

Healthy donor CD34 + cord blood cells were reprogrammed into iPSCs using episomal plasmids encoding OCT4, SOX2, KLF4, CMYC, Lin28, and pEB-Tg as described in detail elsewhere^85^.

### Automated neural cell differentiation

Healthy donor and FSASD iPSCs were differentiated to radial glial cells, cortical neurons, and mature astrocytes as previously described^86,87^. Healthy donor and patient-derived iPSCs were maintained in E8 medium supplemented with CEPT and plated onto vitronectin-coated flasks. For astrocyte differentiation, cells were transitioned to Astro-1 medium (DMEM/F12 with N2, B27 minus vitamin A, 100 nM LDN-193189, and 10 ng/mL each of PDGF-AA, JAGGED-1, DLL-1, ONCOSTATIN M, LIF, and CNTF) to generate radial glial cells (iRGCs). Daily medium changes and sequential passaging were performed to support iRGC expansion. Beginning on Day 15, iRGCs were matured in Astro-2 medium (DMEM/F12 with N2, B27 complete, 1% lipid supplement, and the same panel of recombinant proteins) followed by Astro-3 medium (DMEM/F12 with N2, B27 with vitamin A, 1% lipid supplement, and additional factors including 20 ng/mL hNRG1, 2 µM forskolin, 200 nM phorbol ester, 40 ng/mL T3, and 200 µM ascorbic acid). Cell pellets were collected for downstream analyses at Day 7 (iRGCs), Day 30 (iAs), and Day 50 (iMAs). The entire differentiation process, including media changes and passaging, was automated using the CompacT SelecT system, with manual intervention only during cell dissociation steps.

For cortical neuron differentiation, iPSCs were plated as single cells in CEPT-supplemented E8 medium and transitioned to a neural induction medium (E6 with 100 nM LDN193189 and 2 µM A83-01) at ~ 70% confluency. After 6 days of daily medium changes, cells were dissociated and aggregated into neurospheres using ultralow attachment flasks. Neurospheres were cultured for 2 weeks in DMEM/F12 GlutaMAX medium containing BDNF, GDNF, N2, B27 minus vitamin A, cAMP, and ascorbic acid, and subsequently plated onto Geltrex-coated flasks for an additional 2 weeks of adherent maturation (Day 35). As with the astrocyte protocol, all steps were performed on the CompacT SelecT platform, with only minimal manual handling during dissociation.

Expression of cell type–specific markers confirmed differentiation into the intended neural cell types (Supplementary Fig. 6).

### Quantification of free sialic acid

Neu5Ac, the most abundant mammalian sialic acid^88^, was measured and is referred to as “sialic acid” in this manuscript. Briefly, two million cells per pellet (3 replicates per cell line-cell type) were sonicated in water and 25µL of sonicate was mixed with 25 µL deuterated internal standard and 100µL HPLC-grade water. Samples were filtered using a Spin-X 0.22-mm microcentrifuge filter tube. Free sialic acid was analyzed by UPLC-MS/MS (Waters Acquity I-Class; Xevo TQ-S MS/MS), using a C18 reverse-phase column (Waters Acquity UPLC HSS T3) for chromatographic separation and tandem mass spectrometry operated in selected reaction monitoring mode (electrospray ionization, positive ion mode) for detection. Quantification was accomplished by stable isotope dilution and comparison to a standard curve. Free sialic acid concentrations were normalized by total protein concentration as determined by the Lowry method. All sample runs demonstrated calibration curves with linearity exceeding r^2^ > 0.98.

### Quantification of cellular GSLs

Profiling of glycosphingolipids (GSLs) and glucosylceramide (GlcCer) in cells was performed as detailed previously^89,90^. Three replicates for each cell line-cell type were quantified. Briefly, cellular lipids were extracted overnight at 4 °C using a chloroform–methanol mixture. The GSLs were then purified using C18 columns (Telos). After elution, the GSL fractions were dried down under a stream of nitrogen at 42 °C and subsequently digested with either Cerezyme^®^ (Genzyme) to release glucose from GlcCer or recombinant ceramide glycanase (rEGCase, prepared by Genscript and provided by Orphazyme) to release oligosaccharides from more complex GSLs. The glucose and glycans were then fluorescently labeled with anthranilic acid (2AA). Excess 2AA was removed using DPA-6S SPE columns (Supelco). The purified 2AA-labeled glucose and oligosaccharides were separated and quantified using normal-phase high-performance liquid chromatography (NP-HPLC). A 2AA-labeled glucose homopolymer ladder (Ludger) was included to determine the glucose unit (GU) values for the HPLC peaks. Individual GSL species were identified based on their GU values and quantified by comparing the integrated peak areas with a known amount of 2AA-labeled BioQuant chitotriose standard (Ludger). The results were then normalized to protein content, as determined by the bicinchoninic acid (BCA) assay.

### Quantification of lysosomal hydrolase activities

Lysosomal glycohydrolase activities were measured fluorometrically using synthetic sugar substrates conjugated with the fluorophore 4-methylumbelliferone (4-MU), as described elsewhere^91^. Three replicates for each cell line-cell type were quantified. Neuraminidase (NEU) activity was assessed using two assays: one quantified NEU1/3/4 activity, and the other measured cytosolic NEU2. Both assays utilized 4-MU N-acetylneuraminic acid and were incubated for 4 h at 37 °C. Total β-hexosaminidase and HexA activities were quantified using 4-MU N-acetyl-β-D-glucosaminide. Total Hex assays were incubated for 30 min at 37 °C. HexA assays in iPSCs and iRGCs were incubated for 4 h at 37 °C while iCNs and iIA/iMAs were incubated for 1.5 h at 37 °C. GBA1 and GBA2 activities were measured using 4-MU β-D-glucoside and the samples were incubated for 1 h at 37 °C. NB-DGJ was used to selectively inhibit GBA2 activity, allowing for the determination of GBA1 activity levels^46^. β-galactosidase and α-galactosidase activities were assessed using 4-MU β-D-galactopyranoside and 4-MU α-D-galactoside, respectively, with an incubation time of 30 min at 37 °C. All reactions were terminated with cold 0.5 M Na_2_CO_3_ (pH 10.7), and the released fluorescent 4-MU was quantified using a FLUOstar OPTIMA plate reader (BMG Labtech) with excitation at 360 nm and emission at 450 nm. Enzyme activities were calculated using a standard curve of unconjugated 4-MU and normalized to protein content as measured by BCA assay.

### RNA sequencing and bioinformatic analyses

For bulk RNA sequencing, RNA was extracted using the RNeasy Plus Mini Kit (Qiagen) following the manufacturer’s instructions. The extraction process was automated using the QIAcube workstation (Qiagen). Genomic DNA was removed via the gDNA eliminator column and on-column DNase I treatment (Qiagen). Sequencing libraries were prepared using the KAPA mRNA HyperPrep Kit (Roche) with a Biomek i7 automated liquid handler (Beckman Coulter). Prior to sequencing, the libraries were quantified by qPCR using the KAPA Library Quantification Kit for Illumina platforms (Roche) on a QuantStudio 12 K Flex Real-Time PCR System (Thermo Fisher Scientific). All samples were normalized based on concentration, pooled, and sequenced on the NovaSeq 6000 System (Illumina). Reads were aligned to GRCh38 with HISAT2^92^ and read counts were generated with DESeq2 R package^93^. GSL metabolism genes, including biosynthesis and catabolism genes listed in the pathways depicted in Platt^42^ were examined. Volcano plots were generated using the EnhancedVolcano R package^94^. R version 4.3.1 was used for all analyses.

### Statistical analyses

Statistical analyses were conducted using an unpaired t-test or ordinary one-way ANOVA with Šídák’s multiple comparisons test between healthy donors and FSASD samples using GraphPad Prism (GraphPad, version 10.0.3). Correlations were analyzed with Pearson correlation analysis. A two-sided *p*-value significance was set at 0.05.

## Supplementary Information

Supplementary Information 1.

Supplementary Information 2.

Supplementary Information 3.

## Data Availability

The datasets generated during and analyzed during the current study are available from the corresponding author on reasonable request.
