# Supplementary material for: Lysosomal free sialic acid storage disorder iPSC-derived neural cells display altered glycosphingolipid metabolism
Source: Sci Rep. 2025 Aug 13;15:29708. doi: 10.1038/s41598-025-12682-4 (PMC12350760; doi:10.1038/s41598-025-12682-4)
Supplement: Supplementary file 1 — Supplementary Information 1. [file 41598_2025_12682_MOESM1_ESM.pdf]

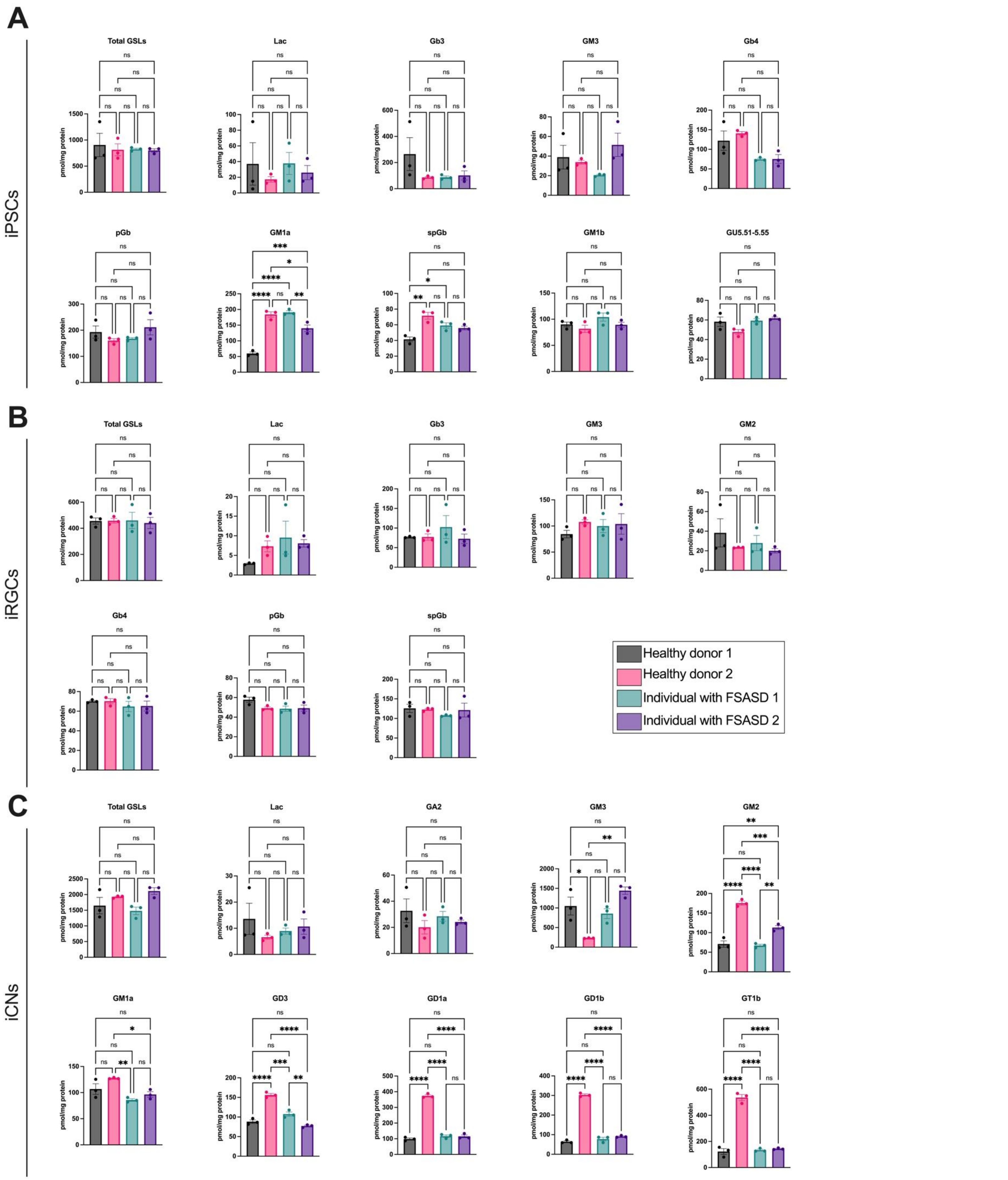

Supplementary Figure 1. Levels of total GSLs and individual GSL species in FSASD and healthy donors in iPSCs, iRGCs, and iCNs by individual study subject. (A) iPSCs, **(B)** iRGCs, and **(C)** iCNs. GSL levels quantified via HPLC were normalized to total protein content of the whole cell lysates. Each point corresponds to one of the three replicates for each cell line. Mean ± SEM; ordinary one-way ANOVA with Šídák's multiple comparisons test with *p*-value <0.05 (*), <0.0099 (**), <0.0009 (***), <0.0001 (****), and ns = not significant.

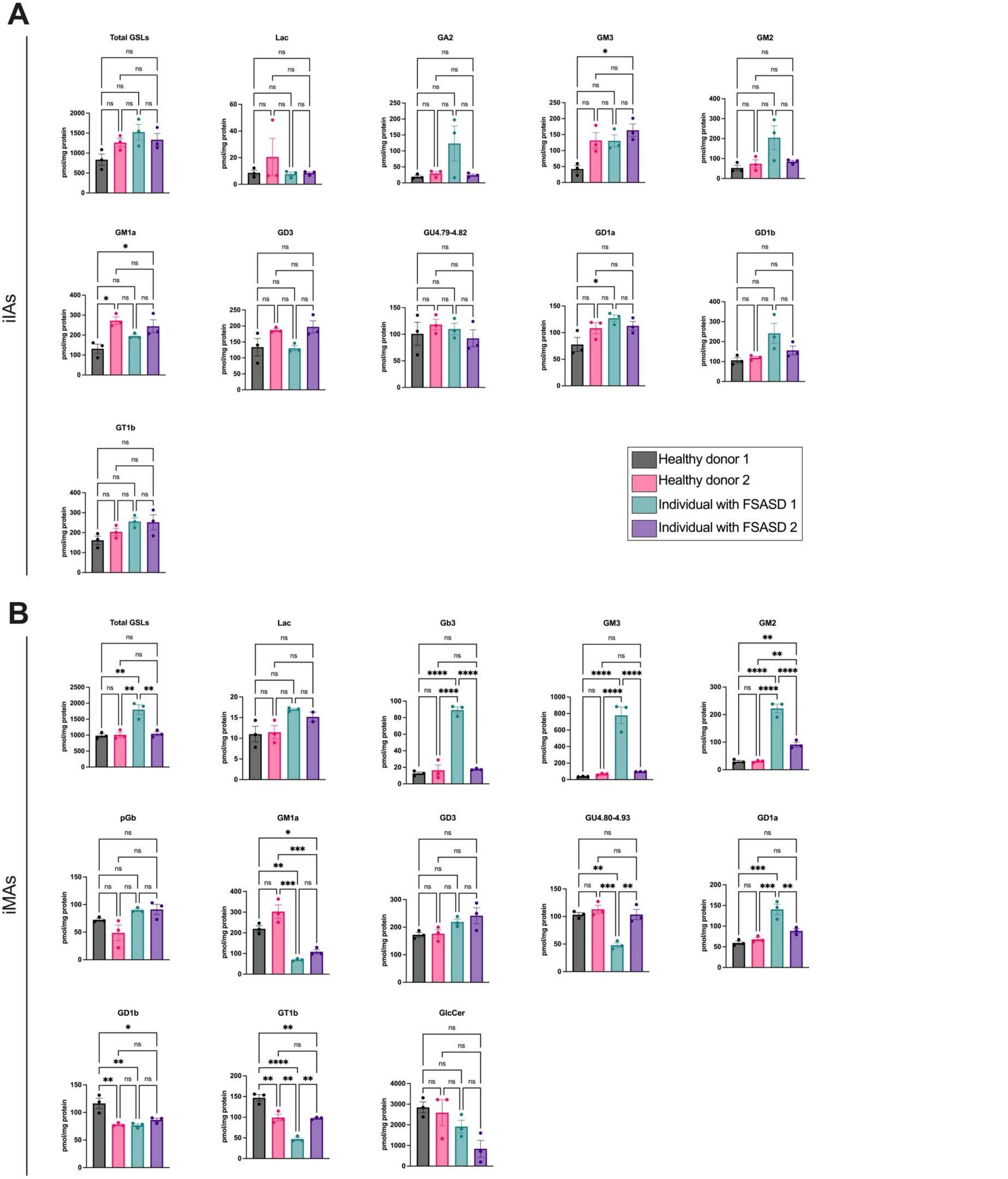

Supplementary Figure 2. Levels of total GSLs and individual GSL species in FSASD and healthy donors in iIAs and iMAs by individual study subject. (A) iIAs and **(B)** iMAs, GSL levels quantified via HPLC were normalized to total protein content of the whole cell lysates. Each point corresponds to one of the three replicates for each cell line. Mean ± SEM; ordinary one-way ANOVA with Šídák's multiple comparisons test with *p*-value <0.05 (*), <0.0099 (**), <0.0009 (***), <0.0001 (****), and ns = not significant.

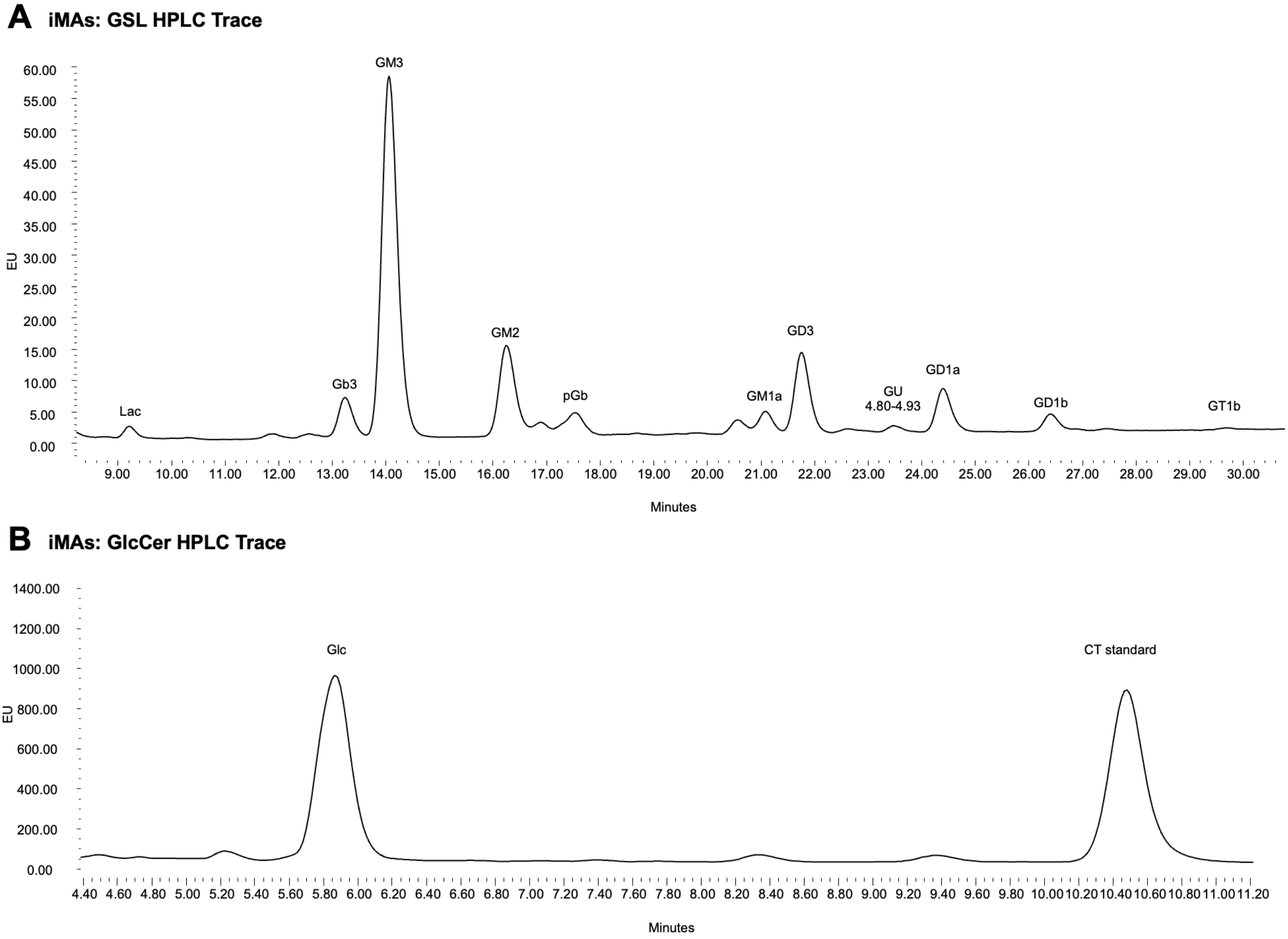

Supplementary Figure 3. Representative HPLC traces of GSLs and glucosylceramide (GlcCer) assays in iMAs. (A) GSL HPLC trace and **(B)** GlcCer HPLC trace annotated with provisionally assigned GSL species.

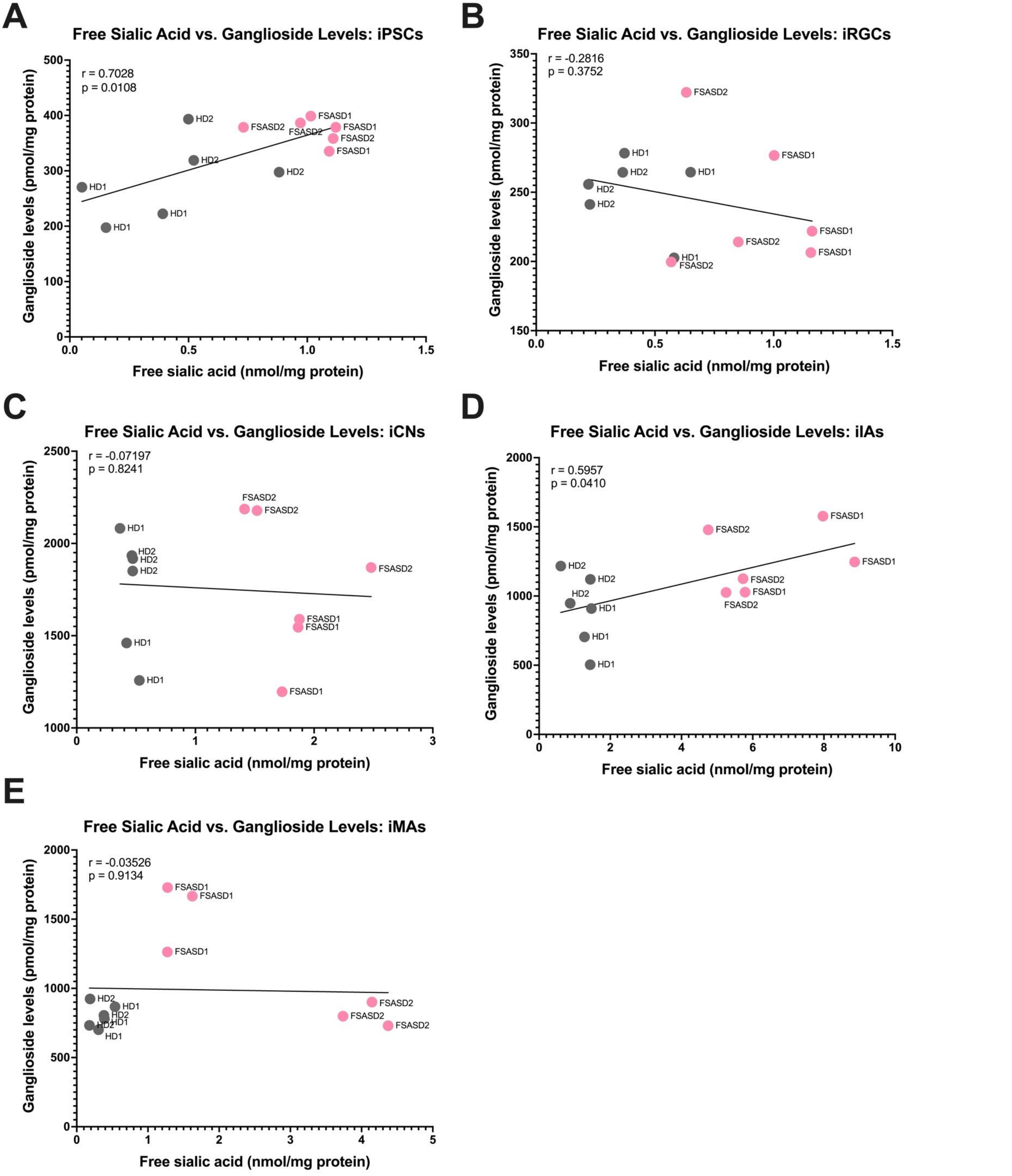

Supplementary Figure 4. Correlation of free sialic levels with levels of gangliosides in each cell type. Free sialic acid levels normalized to total protein content versus ganglioside levels normalized to total protein content in **(A)** iPSCs, **(B)** iRGCs, **(C)** iCNs, **(D)** iIAs, and **(E)** iMAs. Three replicates per cell line as annotated. Data were analyzed using Pearson correlation analysis with r and *p*-values as indicated in each panel. HD = healthy donor.

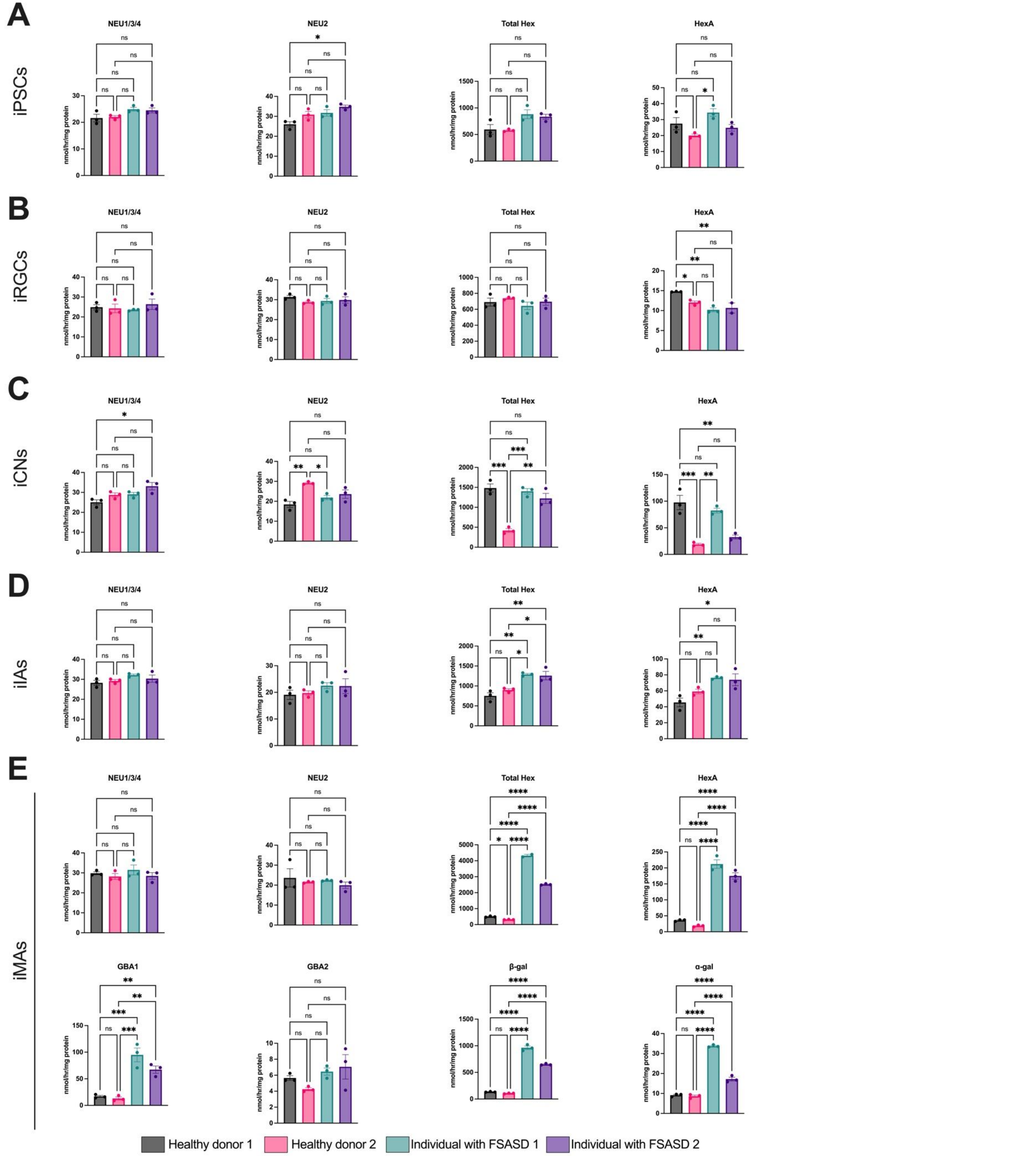

Supplementary Figure 5. Activity levels of GSL hydrolases in the neural cell types by individual study subject. (A) iPSCs, **(B)** iRGCs, **(C)** iCNs, **(D)** iIAs, and **(E)** iMAs. The following enzyme activities were measured via 4-MU-based assays in all cell types (using whole cell lysates): neuraminidase (NEU1/3/4 and NEU2) and β-hexosaminidase (total Hex and HexA). Additional enzymes were examined in iMAs including glucocerebrosidase (GBA1 and GBA2), β-galactosidase (β-gal), and α-galactosidase (α-gal). Activity represented as nmol per hour per mg total protein content. Each point corresponds to one of the three replicates for each cell line. Mean ± SEM; ordinary one-way ANOVA with Šídák's multiple comparisons test with *p*-value <0.05 (*), <0.0099 (**), <0.0009 (***), <0.0001 (****), and ns = not significant.

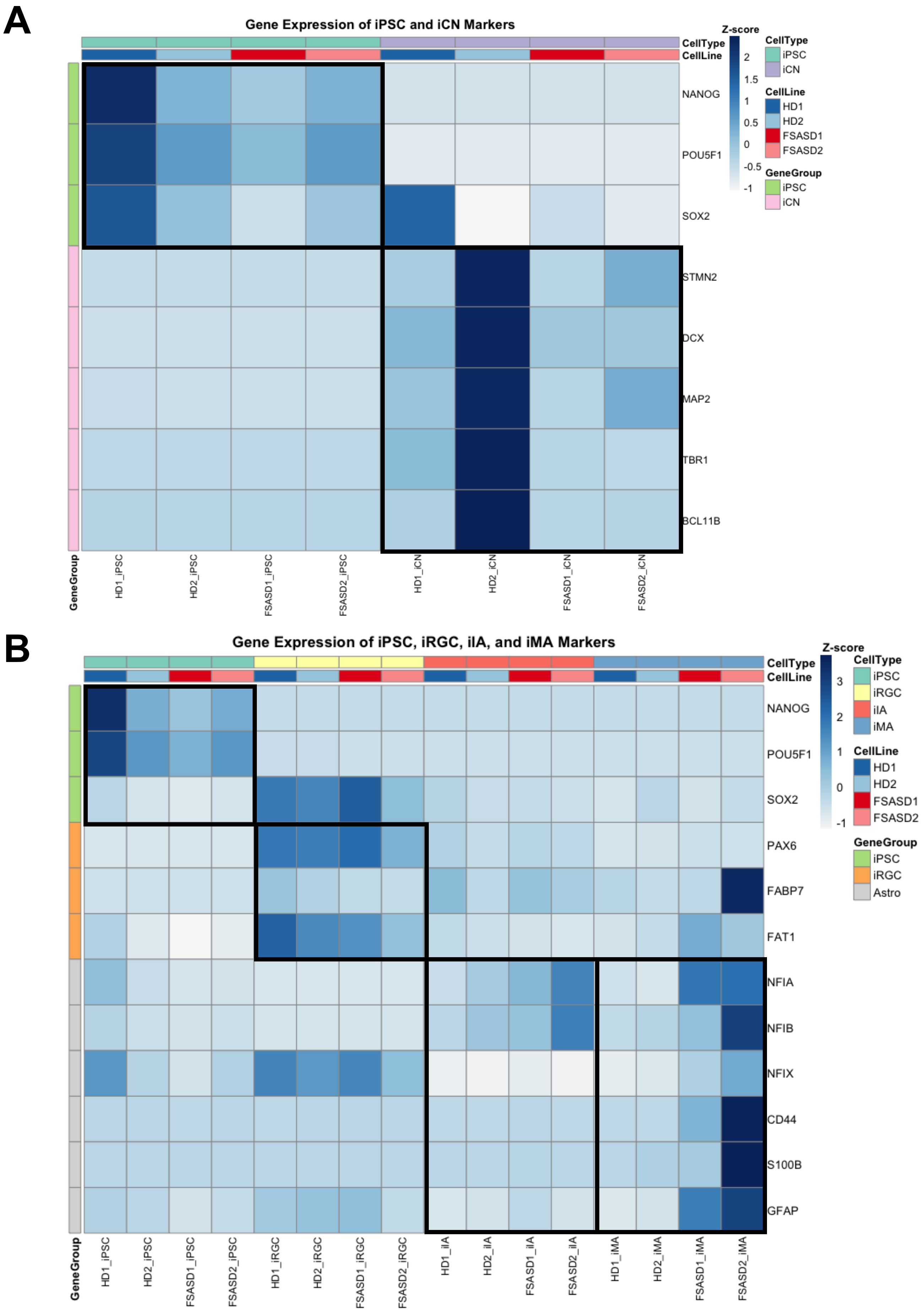

Supplementary Figure 6. Transcriptomic profiling of cell type-specific markers across differentiated neural lineages. (A) Heatmap of z-score–normalized expression values for canonical marker genes of induced pluripotent stem cells (iPSCs) and cortical neurons (iCNs). **(B)** Heatmap of z-score–normalized expression for marker genes associated with iPSCs, induced radial glial cells (iRGCs), immature astrocytes (iIAs), and mature astrocytes (iMAs). Columns represent the mean expression across three biological replicates for each cell line and cell type. HD = healthy donor.

Supplementary Table 1. Summary of cell lines included in study

**Cell line**	**Sex**	**Age (years)**[1]	**Race/ Ethnicity**	**Disease**	***SLC17A5* genotype**[2] **(allele 1/allele 2)**	**Cell source**	**iPSC reprogramming method**
Healthy donor 1	Male	0	Unknown	NA	Wild-type	CD34+ cord blood	Episomal plasmid
Healthy donor 2	Male	0	Unknown/ Hispanic or Latino	NA	Wild type	CD34+ cord blood	Episomal plasmid
Individual with FSASD 1	Female	1.3	Multiple race/ Hispanic or Latino	Intermediate -severe FSASD	c.406A>G; p.Lys136Glu/ c.533delC; p.Thr178Asnfs*34	Dermal fibroblasts	Episomal plasmid
Individual with FSASD 2	Male	4	Caucasian/ Finnish	Mild FSASD	c.115C>T; p.Arg39Cys/ c.115C>T; p.Arg39Cys	Dermal fibroblasts	Episomal plasmid

[1]Age of individual at sample collection. [2]Reference sequence based on NM_012434.5. FSASD, free sialic acid storage disorder; NA, not applicable.
